# Association of Physician Peer Influence With Subsequent Physician Adoption and Use of Bevacizumab

**DOI:** 10.1001/jamanetworkopen.2019.18586

**Published:** 2020-01-03

**Authors:** Nancy L. Keating, A. James O’Malley, Jukka-Pekka Onnela, Stacy W. Gray, Bruce E. Landon

**Affiliations:** 1 Department of Health Care Policy, Harvard Medical School, Boston, Massachusetts; 2 Division of General Internal Medicine, Brigham and Women’s Hospital, Boston, Massachusetts; 3 Department of Biomedical Data Science and The Dartmouth Institute for Health Policy and Clinical Practice, Geisel School of Medicine, Hanover, New Hampshire; 4 Department of Biostatistics, Harvard T.H. Chan School of Public Health, Boston, Massachusetts; 5 Department of Population Sciences and Medical Oncology, City of Hope Medical Center, Duarte, California; 6 Division of General Medicine, Beth Israel Deaconess Medical Center, Boston, Massachusetts

## Abstract

**Importance:**

Understanding adoption of new cancer therapies may help identify opportunities to increase use for high-value indications.

**Objective:**

To determine whether use of bevacizumab in 2005 to 2006 by oncologists’ peers was associated with greater bevacizumab use among oncologists in 2007 to 2010.

**Design, Setting, and Participants:**

This cohort study of physicians and their patients took place in 51 randomly selected hospital referral regions in the United States. Participants were 44 012 fee-for-service Medicare beneficiaries aged 65 years or older with cancers of the colorectum, lung, breast, kidney, brain, or ovary treated by 3261 oncologists in 2005 to 2010 and assigned to one of 252 communities. Data were analyzed in 2017 to 2018.

**Exposures:**

Among patients treated with chemotherapy during 2007 to 2010 by an oncologist who had not treated patients with bevacizumab in 2005 to 2006, models assessed the association of bevacizumab use with rates of bevacizumab use in their physician’s community of connected physicians in 2005 to 2006. Models adjusted for patient and physician characteristics and physician, practice, and community random effects.

**Main Outcomes and Measures:**

Receipt of bevacizumab.

**Results:**

A total of 34 750 patients (14 126 [40.6%] aged ≥75 years; 21 321 [61.4%] female) with cancers of the colorectum, lung, breast, kidney, brain, and ovary were treated with chemotherapy in 2005 to 2006 in the 51 hospital referral regions. Among 9262 patients treated in 2007 to 2010 by 829 physicians whose patients did not use bevacizumab in 2005 to 2006, 3654 (39.5%) were aged 75 years or older and 6227 (67.2%) were female. The rate of bevacizumab use relative to other chemotherapy in 2007 to 2010 by tertile of use (bevacizumab for <4.4%, 4.4%-6.2%, and >6.2% of all patients receiving chemotherapy) among their physician’s peers in 2005 to 2006 was 10.0%, 9.5%, and 13.6%, respectively. After adjustment, use of bevacizumab in 2007 to 2010 was greater among physicians in communities with the highest rates of bevacizumab use in 2005 to 2006 compared with those whose peers were in the lowest tertile of bevacizumab use in 2005 to 2006 (adjusted odds ratio, 1.64; 95% CI, 1.20-2.25).

**Conclusions and Relevance:**

This study found that an increase in oncologists’ adoption and use of bevacizumab in the years after its approval was associated with their peer physicians being earlier adopters. As organizations seek to provide better care at lower costs, interventions that leverage physician ties may help to promote adoption of high-value use of new cancer treatments and deimplementation of low-value therapies.

## Key Points

**Question:**

Is peer influence associated with physician adoption of new cancer therapies?

**Findings:**

In this cohort study of 44 012 Medicare beneficiaries with cancer treated by 3261 oncologists, use of bevacizumab in 2007 to 2010 was greater among physicians whose peers had the highest rates of bevacizumab use in 2005 to 2006 compared with those whose peers were in the lowest tertile of bevacizumab use.

**Meaning:**

These findings suggest that interventions that leverage physician ties have potential to promote adoption of high-value use of new cancer treatments.

## Teaser

This cohort study examines the association of physician peer use of bevacizumab with subsequent physician adoption and use of bevacizumab to treat US Medicare beneficiaries with cancer.

## Introduction

Adoption and diffusion of costly new technologies is of interest to health systems in high-resource countries, but in particular for policy makers in the United States, where health care spending accounts for 18% of the gross domestic product. Substantial variation in use of new technologies suggests that factors beyond patient clinical characteristics are driving adoption and use. Novel systemic therapies to treat cancer are of particular interest both because of the rapidly growing number of anticancer medications and their high cost. The US Food and Drug Administration (FDA) approved indications for 91 cancer drugs during 2014 to 2018,^1^ and prices of newly approved cancer drugs are increasing substantially faster than inflation.^2^ Although some of these drugs have had a sizable impact on cancer outcomes, most have modest benefits, and evidence suggests that prices may not correlate strongly with benefit.^3,4^ In addition, cancer drugs are frequently used off label,^5^ where benefits may be less clear. Thus, understanding adoption of new cancer therapies may help us to better understand what factors might increase the appropriateness of their use.

Bevacizumab, one of the first widely used biological cancer therapies, was initially approved by the FDA in 2004 for the treatment of colorectal cancer, and since then it has been approved for treating advanced-stage cancers of the lung, breast, brain, kidney, cervix, and ovary. Prior research suggests relatively rapid diffusion of bevacizumab across oncology practices with substantial variation in use across practices.^6^ This variation, however, remains largely unexplained.

Social influence is thought to be an important driver of the adoption of new medical therapies by physicians. In prior work, we applied social networks analysis to demonstrate that information-sharing connections among physicians can be identified using patient-sharing relationships observed in Medicare data.^7,8,9^ Using this approach, we recently demonstrated that characteristics of physicians’ social networks and the positions of physicians in the networks are associated with overall spending and use of services for Medicare beneficiaries.^10^ In addition, we documented that connected physicians may influence the intensity of end-of-life care delivered to patients with cancer.^11^ Other evidence suggests that a physician’s propensity to incorporate novel imaging technologies in the care of patients with breast cancer is associated with the early adoption of these technologies by other physicians with whom they are connected.^12^

In this article, we applied network analysis methods to understand adoption of bevacizumab by oncologists for patients with cancer. Specifically, we identified physicians and communities (clusters) of physicians with early use of bevacizumab in 2005 and 2006 (the billing code for bevacizumab was made available in 2005 following its first approval in 2004). Then, focusing on physicians who did not use bevacizumab when treating patients with cancer with chemotherapy in the 2005 to 2006 period, we assessed the extent to which use of bevacizumab by a physician’s community of connected peer physicians in the earlier 2005 to 2006 period was associated with the physician’s use in the later 2007 to 2010 period. We hypothesized that patients of physicians whose peers had greater use of bevacizumab in 2005 to 2006 would be associated with increased use of bevacizumab in the later period.

## Methods

### Data and Participants

We used Medicare data from 2005 to 2010 for 100% of Medicare fee-for-service beneficiaries aged 65 years or older and living in 50 randomly sampled (probability proportional to size) hospital referral regions (HRRs) in the United States.^8,9,10^ We also included patients in the Boston HRR because of our familiarity with it. We identified individuals with cancer based on physician visits with a diagnosis code for cancer (eAppendix 1 in the Supplement). A total of 44 012 Medicare beneficiaries with cancer treated by 3261 oncologists in 51 HRRs were identified.

We identified patients with cancers of the colorectum, lung, breast, kidney, brain, or ovary treated with chemotherapy during 2005 to 2010.^6^ These are cancers for which bevacizumab has been approved for use since 2004. We then assigned patients to physicians based on the specialty of the physicians they saw, prioritizing visits to oncologists over visits to other types of physicians (eAppendix 2 in the Supplement). The study was approved by the Harvard Medical School Committee on Human Studies. Because the study used data that were previously collected for other purposes, a waiver of informed consent was granted. Analyses for this study were conducted in 2017 to 2018. This article follows Strengthening the Reporting of Observational Studies in Epidemiology (STROBE) reporting guideline for cohort studies.

### Assignment of Physicians to Networks and Communities

As described previously, we used data for all fee-for-service Medicare patients in the 51 HRRs to define networks of physicians based on shared patients.^8,13^ We previously demonstrated that physicians who share patients in this manner are likely to have information-sharing relationships.^7^ We excluded claims for specialties in which individual physicians are not selected (eg, anesthesia, radiology), nondirect patient care specialties, and laboratory and other services that do not require a physician visit. We identified all evaluation and management services for outpatient and inpatient visits and for procedures with a relative value unit value of 2.0 or greater (to capture surgical procedures that may be reimbursed via bundled payments that include preoperative and postoperative office visits). After excluding any physician seeing fewer than 30 Medicare patients in a year or whose zip code was outside of the HRR, we constructed physician networks based on claims, identifying physicians pairs who shared patients with each other in 2005 to 2006 (baseline period) within an episode of cancer care (defined using Optum’s Episode Treatment Group software).^10,14,15,16^ Focusing on visits within episodes of cancer care^17^ allowed us to identify physicians who were addressing a patient’s cancer. See the eFigure in the Supplement for additional details about the network construction.

Following other research,^12^ we sought to characterize use of bevacizumab among oncologists in a community or cluster of connected physicians in 2005 to 2006. We thus identified discrete communities of physicians within each HRR who were more likely to share the care of a group of patients with cancer using the generalized Louvain method community detection algorithm (version 2).^18^ We assigned physicians to communities based on care patterns in the baseline period (2005-2006).

### Bevacizumab Use in the Baseline Period

We studied bevacizumab because it was one of the first widely used biological therapies for cancer, it was used for patients with a variety of cancer types, and its diffusion across oncology practices was relatively rapid during the period of our study (2005-2010).^6^ Although bevacizumab was first approved by the FDA for treatment of colorectal cancer in 2004, the procedure code specific to bevacizumab was not available until 2005. The colorectal cancer indication was expanded in 2006 and 2013; additional approvals for lung cancer (2006), breast cancer (2008, later withdrawn), glioblastoma (2009), renal cell cancer (2009), cervical cancer (2014), and ovarian cancer (2014) followed. Diffusion of bevacizumab among Medicare patients typically started before the approval dates and was most rapid for colorectal cancer, followed by lung and breast cancer, until the 2009 approval for glioblastoma, which resulted in rapid adoption.^6^

We identified 34 750 patients treated by 2432 physicians providing chemotherapy to at least 20 patients with cancers of the colorectum, lung, breast, kidney, brain, or ovary in 2005 to 2006 who were assigned to one of 252 communities with at least 2 eligible physicians providing chemotherapy. We characterized the proportion of all patients with the cancers of interest in each network community who were undergoing chemotherapy and who received bevacizumab at any time during 2005 to 2006. Bevacizumab use in the baseline period (2005-2006) across communities ranged from 0% to 31.5% of all patients receiving chemotherapy for the eligible cancers (median [interquartile range], 8.2% [5.8%-11.0%]). We expected rates of bevacizumab use to be relatively low because we lacked clinical information about stage, histology, and prior treatments that might determine ideal candidates for this treatment and because bevacizumab was approved for some cancers we studied after 2006. In preliminary analyses, associations of community-level use in the baseline period with new use in 2007 to 2010 was nonlinear; therefore, we categorized community-level use into tertiles (bevacizumab for <4.4% of all patients receiving chemotherapy, for 4.4%-6.2% of all patients receiving chemotherapy, and for >6.2% of all patients receiving chemotherapy).

### Bevacizumab Use in the Follow-up Period

We characterized bevacizumab use during 2007 to 2010 among patients of physicians who prescribed chemotherapy during 2005 to 2010 but did not use bevacizumab in 2005 to 2006. The unit of analysis was the patient-year, and we assessed use of bevacizumab by patients at any point during a year in which they received any chemotherapy.

### Control Variables

For each patient, we characterized age, sex, race/ethnicity, year, and cancer site (lung, colorectal, breast, kidney, ovary, or brain based on *International Classification of Diseases, Ninth Revision [ICD-9] *codes). We used hierarchical condition category scores for case mix adjustment.^19^ Race/ethnicity was reported based on Medicare administrative files; it was included because other research suggests differences in cancer treatments by race/ethnicity. Variables are categorized as in Table 1, except for patient age and physician age, which were included as continuous variables in models. We characterized physician age and sex based on linkage with the American Medical Association Physician Masterfile. We assigned physicians to practices based on tax identification numbers based on the plurality of evaluation and management visits with patients. We characterized practice size based on the number of physicians billing for chemotherapy from a practice in a given year. See eAppendix 3 in the Supplement for additional details.

**Table 1.** Characteristics of Patients, Physicians, and Practices

Characteristic	No. (%)	
	Early Adoption Period: 2005-2006	Late Adoption Period: 2007-2010
Patient characteristics		
Patients, No.	34 750	9262
Age, y		
65-74	20 624 (59.3)	5608 (60.5)
75-84	12 641 (36.4)	3149 (34.0)
≥85	1485 (4.3)	505 (5.5)
Sex		
Male	13 429 (38.6)	3035 (32.8)
Female	21 321 (61.4)	6227 (67.2)
Race/ethnicity		
White	30 667 (88.3)	7902 (85.3)
Black	2837 (8.2)	946 (10.2)
Hispanic	539 (1.5)	172 (1.9)
Other or unknown	707 (2.0)	242 (2.6)
Cancer site		
Lung	14 821 (42.6)	3651 (19.4)
Colorectal	9089 (26.2)	1461 (15.8)
Breast	7163 (20.6)	2151 (23.2)
Kidney	572 (1.7)	292 (3.2)
Ovary	2990 (8.6)	1595 (17.2)
Brain	115 (0.3)	112 (1.2)
Hierarchical condition category score, mean (SD)	3.64 (1.73)	3.95 (1.86)
Year		
2005	13 963 (40.2)	NA
2006	20 787 (59.8)	NA
2007	NA	2918 (31.5)
2008	NA	2374 (25.6)
2009	NA	2011 (21.7)
2010	NA	1959 (21.2)
Physician characteristics		
No. of oncologists	2432	829
No. of patients per physician		
Mean (SD)	14.6 (18.5)	11.2 (16.5)
Median (IQR)	5 (1-22)	4 (1-13)
Physician age, mean (SD), y	49.4 (9.7)	49.7 (10.1)
Physician sex		
Male	1905 (78.3)	617 (74.3)
Female	506 (20.8)	205 (24.7)
Missing	21 (0.9)	<11 (<1.0)
Practice characteristics		
No. of practices	1031	440
No. of oncologists per practice		
1	725 (70.3)	327 (74.3)
2	100 (9.7)	52 (11.8)
≥3	206 (20.0)	61 (13.9)
No. of physicians per practice, median (IQR)	1 (1-2)	1 (1-2)
No. of patients per practice		
Mean (SD)	34.0 (75.7)	21.1 (36.6)
Median (IQR)	4 (1-31)	6 (1-26)

Abbreviations: IQR, interquartile range; NA, not applicable.

### Statistical Analysis

We used patient-year level models with physician, practice, and community random effects to assess bevacizumab use in 2007 to 2010 as a function of use of bevacizumab in 2005 to 2006 in a physician’s community. The population of interest included all patients receiving chemotherapy in 2007 to 2010 who were receiving chemotherapy from physicians who did not use bevacizumab in 2005 to 2006; the dependent variable was the patient’s use of bevacizumab during 2007 to 2010 received from physicians who did not treat patients with bevacizumab in 2005 to 2006. The independent variables of interest were the tertiles of the adjusted proportion of patients who were treated with bevacizumab in 2005 to 2006 in the community to which the patient’s physician was assigned, and we adjusted for the control variables described. We compared the middle and top tertiles with the lowest tertile of 2005 to 2006 bevacizumab use. Models adjusted for patient age, sex, race/ethnicity, cancer type, comorbidity, year, physician age, sex, and practice size and included physician, practice, and community random effects. Two-sided *P* values less than .05 were considered statistically significant. Missing data on covariates were minimal; where present (patient race/ethnicity and physician sex), they were analyzed as a separate category as in Table 1.

### Sensitivity Analyses

The 829 physicians in the 2007 to 2010 cohort were assigned to 432 practices; 405 of these practices (92.3%) were each assigned to a single community, 24 practices (5.5%) were in 2 communities, and the remaining 10 practices (2.3%) were assigned to 3 or more communities. In sensitivity analyses limiting the cohort to patients in the practices assigned to a single community, results were similar and are not presented. In addition, as described, our analyses used patient-year observations; 22% of patients received chemotherapy in 2 years, 4% in 3 years, and 1% in all 4 years. Our models with physician random effects did not allow us to individually adjust for clustering of patients with more than 1 observation. Accounting for repeated measurements on the same patient would likely have minimal impact because most patients contributed a single observation. In sensitivity analyses focusing on the first observation for a patient only, results were similar and are not presented. In additional sensitivity analyses, we assessed whether community size was associated with bevacizumab use in 2007 to 2010 and whether the association of bevacizumab use by peers in 2005 to 2006 with use in 2007 to 2010 varied by community size (the inclusion of these variables evaluates whether the association of peer bevacizumab use interacts with community size*)*. We found no associations and thus did not include these variables in final models.

## Results

We identified 34 750 patients (14 126 [40.6%] aged ≥75 years; 21 321 [61.4%] female; 14 821 [42.6%] with lung cancer) with cancers of the colorectum, lung, breast, kidney, ovary, and brain treated with chemotherapy during 2005 to 2006 in the 51 HRRs. We also identified 9262 patients (3654 [39.5%] aged ≥75 years; 6227 [67.2%] female) with these cancers treated with chemotherapy during 2007 to 2010 by a physician who had not treated patients with bevacizumab in 2005 to 2006. These patients were treated by 829 physicians billing under 440 tax identification numbers and assigned to 193 communities (mean [SD] of 294 [213] patients per community [range, 24-1175] and 22 [21] physicians per community [range, 3-172]). Characteristics of the patients are included in Table 1. Patients treated with chemotherapy in 2007 to 2010 who were treated by physicians who did not adopt bevacizumab in 2005 to 2006 had a similar distribution of characteristics to the 2005 to 2006 cohort, except that they were more likely to have breast or ovarian cancer, consistent with the later FDA approval of bevacizumab for these indications. The physicians and practices of the 2007 to 2010 cohort also tended to see fewer patients than physicians in the 2005 to 2006 cohort.

In unadjusted analyses, use of bevacizumab relative to all other chemotherapy in our cohort in 2007 to 2010 was 10.0% among patients of physicians whose peers in their community had bevacizumab use rates in 2005 to 2006 in the lowest tertile (<4.4% of patients) (Table 2). This rate was 9.5% among patients of physicians whose peers in their community had bevacizumab use rates in the middle tertile in 2005 to 2006 (4.4%-6.2% of patients) and 13.6% among patients of physicians whose community bevacizumab use in 2005 to 2006 was in the highest tertile (>6.2% of patients).

**Table 2.** Use of Bevacizumab in 2007 to 2010 Based on Peers’ Use of Bevacizumab in 2005 to 2006

Characteristic	Unadjusted % of Patients Receiving Bevacizumab in 2007-2010	Adjusted Odds Ratio for Bevacizumab Receipt in 2007-2010 (95% CI)^a^	*P* Value
Patients of physician whose peers used bevacizumab in 2005-2006, %			
<4.4	10.0	1 [Reference]	
4.4-6.2	9.5	1.05 (0.78-1.40)	.77
>6.2	13.6	1.64 (1.20-2.25)	.002
Patient age, continuous, per y	NA	0.98 (0.97-0.99)	<.001
Patient age categories, y			
65-74	11.4	NA	
75-84	9.7	NA	
≥85	9.3	NA	
Sex			
Female	9.3	1 [Reference]	
Male	13.6	0.94 (0.81-1.09)	.39
Race/ethnicity			
White	10.9	1 [Reference]	
Black	7.9	0.88 (0.69-1.11)	.26
Hispanic	14.5	1.26 (0.90-1.94)	.31
Other or unknown	11.6	0.93 (0.65-1.33)	.69
HCC score, continuous^b^		1.03 (0.99-1.06)	.16
HCC score categories^b^			
≤2.868	9.7	NA	
>2.868 to ≤4.251	11.2	NA	
>4.251	11.1	NA	
Cancer site			
Lung	8.0	1 [Reference]	
Colorectal	26.0	4.91 (4.17-5.79)	<.001
Breast	6.7	0.80 (0.65-1.00)	.045
Kidney	6.5	0.83 (0.52-1.31)	.42
Ovary	5.4	0.95 (0.73-1.23)	.68
Brain	63.4	18.03 (10.89-29.85)	<.001
Year			
2007	9.1	1 [Reference]	
2008	9.9	1.34 (1.12-1.60)	.002
2009	11.5	1.67 (1.39-2.00)	<.001
2010	13.3	2.11 (1.76-2.53)	<.001
Physician age, continuous, per y	NA	0.99 (0.98-1.00)	.01
Physician age categories, y			
≤42.6	13.1	NA	
42.7 to ≤52.4	10.4	NA	
>52.4	8.7	NA	
Physician sex			
Male	11.1	1 [Reference]	
Female	10.4	1.01 (0.80-1.26)	.94
No. of physicians in practice			
1	12.0	1 [Reference]	
2	10.1	0.83 (0.60-1.16)	.27
≥3	9.7	1.04 (0.79-1.35)	.79

Abbreviations: HCC, hierarchical condition categories; NA, not applicable.

^a^ Using logistic regression with random physician, practice, and community effects, adjusted for all variables in the table.

^b^ The HCC scores assess comorbidity as well as the severity of the cancer, with cancers for which codes reflecting metastatic cancer are used receiving higher HCC scores.

Table 2 shows results from the models estimating use of bevacizumab in 2007 to 2010 and incorporating physician, practice, and community random effects. Use of bevacizumab in 2007 to 2010 was similar for patients of physicians whose peers in their community had bevacizumab use in 2005 to 2006 in the middle tertile compared with the lowest tertile in 2005 to 2006 (adjusted odds ratio, 1.05; 95% CI, 0.78-1.40). However, among patients of physicians whose peers had use of bevacizumab in 2005 to 2006 in the highest tertile, use of bevacizumab in 2007 to 2010 was statistically higher than among patients of physicians whose peers were in the lowest tertile of bevacizumab use in 2005 to 2006 (adjusted odds ratio, 1.64; 95% CI, 1.20-2.25). Bevacizumab use was also more frequent among younger vs older patients, individuals with colorectal or brain cancers vs lung or breast cancers, and patients of younger vs older physicians (Table 2).

## Discussion

We examined adoption of bevacizumab in 2007 to 2010 among oncologists who had not prescribed this biological therapy drug in 2005 to 2006 and found that having a community of connected peer oncologists with greater use of bevacizumab in 2005 to 2006 was associated with increased use of bevacizumab in 2007 to 2010, even after accounting for practice-level influences. Other evidence has shown substantial variation in use of bevacizumab across oncology practices,^6^ but the sources of these differences are not well understood.

Our findings are consistent with another study^12^ documenting the importance of peer influence on adoption of new imaging technologies in the care of patients with breast cancer. That study identified surgeons who were not early adopters of magnetic resonance imaging and positron emission tomography in the perioperative setting for women with newly diagnosed breast cancer. They demonstrated that belonging to a peer group of physicians who ordered more of these imaging tests in the baseline period was associated with physician adoption of these tests during the follow-up period. In a related analysis,^20^ they also found that direct connection with early adopters of magnetic resonance imaging was associated with greater surgeon use of this technology, and the strength of the association attenuated as the degree of separation between the 2 physicians increased. Another recent study^21^ underscored the role of physician peers on adoption of 3 first-in-class oral medications (dabigaltran, sitagliptin, and aliskiren), finding that the use of these drugs by peers as measured by patient-sharing networks was more important than prescribing by peers within the hospitals or medical groups where they practiced. This may suggest that exposure to these new drugs prescribed by another physician increases knowledge about the drugs and comfort prescribing them.

Rogers’^22^ theory of diffusion of innovation describes diffusion as the process by which an innovation is communicated over time among the participants in a social system. Research by Coleman et al^23^ focused on diffusion of medical technologies, finding that physicians were often reluctant to adopt new medical technologies until they saw their colleagues using them. Our study expands on this by characterizing use of bevacizumab within a community of physicians, suggesting an important role of social reinforcement among physicians, rather than just the communication about a medical innovation from a colleague. This suggests that the adoption of bevacizumab is a complex contagion (ie, needing social reinforcement) rather than a simple contagion (ie, needing simple informational contact only).^24,25^

With increasing efforts to ensure that physicians deliver high-value care, opportunities to influence physician prescribing decisions are of interest to policy makers and others seeking to improve the value of care. This is particularly true with the proliferation of new oncology therapies, many of which have very high prices and modest or limited benefits.^3,4^ Current efforts to promote delivery of high-value oncology care, such as the Oncology Care Model^26^ and other alternative payment models, target physician organizations, a strategy that, in part, relies on the development of practice norms that arise in response to these incentives. Such initiatives are rooted in the expectation that individual physicians can influence the practice of their peers. However, physician behavior is quite challenging to change, and relatively few studies have demonstrated the ability of physicians to directly influence clinical decisions of others. Moreover, understanding which physicians to target is also important. For example, academic detailing has potential to influence clinician behavior, especially if targeted to key opinion leaders who are perceived as influential experts by their peers.^21,27^ Other evidence suggests that in decentralized networks, social influence can generate learning dynamics that improve the performance of the group,^28^ which could potentially slow adoption of low-value therapies or those lacking sufficient evidence.

Consistent with a prior report, we observed less use of bevacizumab for older patients and individuals with breast, kidney, and ovarian cancer (vs colorectal cancer and cancers of the brain). These findings are consistent with evidence for maximizing the ratio of benefits vs harms. We also observed less use among older physicians. Other research has identified practice characteristics associated with adoption of bevacizumab for Medicare patients (ie, independent vs academic practices, practices with a greater ratio of oncologists to other physicians).^6^ Our use of physician, practice, and community random effects allowed us to look within practices and geographically distributed communities of physicians to understand specifically the association of peer influence with use of bevacizumab while simultaneously accounting for unexplained variation at the physician, practice, and community levels.

### Strengths and Limitations

This study had several strengths, including the large population of patients and physicians studied, the ability to examine a treatment as it was being adopted, and the longitudinal study design. The latter allowed us to demonstrate evidence of an association over time, rather than cross-sectional associations of similar care patterns, which might be explained by homophily—the likelihood that physicians tend to share patients with other physicians who are similar to them.^8^ Our study also has limitations. We studied bevacizumab use among older individuals enrolled in fee-for-service Medicare who received infused chemotherapy in 2006 to 2010; the generalizability of our findings to younger patients and those treated with oral cancer therapies requires further study. Also, our random sample of 100% Medicare data for 50 HRRs was supplemented with 1 additional HRR that was not randomly sampled. In addition, we used administrative data to identify physician peer groups rather than asking physicians directly; however, prior work^7^ has shown that administrative data can provide a valid measure of connected physicians. The administrative data also lacked detailed information on cancer stage that might have allowed us to better identify candidates for treatment.

## Conclusions

In conclusion, oncologists’ adoption and use of bevacizumab in the years after its approval was greater if their peer physicians were earlier adopters. As organizations seek to provide better care at lower costs, interventions that leverage physician ties may help to promote adoption of high-value use of new cancer treatments and deimplementation of low-value therapies.

## Notes

Supplement. **eAppendix 1.** Codes for Identifying Cancer Patients Based on Physician Visits and Cancer Diagnosis and Chemotherapy Use **eAppendix 2.** Assignment of Patients to Physicians **eFigure.** A Bipartite Network Used to Construct a Unipartite Network **eAppendix 3.** Assignment of Physicians to Practices
