# Supplementary material for: Association of Physician Peer Influence With Subsequent Physician Adoption and Use of Bevacizumab
Source: JAMA Netw Open. 2020 Jan 3;3(1):e1918586. doi: 10.1001/jamanetworkopen.2019.18586 (PMC6991243; doi:10.1001/jamanetworkopen.2019.18586)
Supplement: Supplement. — eAppendix 1. Codes for Identifying Cancer Patients Based on Physician Visits and Cancer Diagnosis and Chemotherapy Use eAppendix 2. Assignment of Patients to Physicians eFigure. A Bipartite Network Used to Construct a Unipartite Network eAppendix 3. Assignment of Physicians to Practices [file jamanetwopen-3-e1918586-s001.pdf]

Supplementary Online Content

Keating NL, O'Malley AJ, Onnela J-P, Gray SW, Landon BE. Association of physician peer influence with subsequent physician adoption and use of bevacizumab. *JAMA Netw Open*. 2020;3(1):e1918586. doi:10.1001/jamanetworkopen.2019.18586

eAppendix 1. Codes for Identifying Cancer Patients Based on Physician Visits and Cancer Diagnosis and Chemotherapy Use
eAppendix 2. Assignment of Patients to Physicians
eFigure. A Bipartite Network Used to Construct a Unipartite Network
eAppendix 3. Assignment of Physicians to Practices

This supplementary material has been provided by the authors to give readers additional information about their work.

© 2020 Keating NL et al. *JAMA Network Open.*

Appendix. Identification of patients and assignment of patients to physicians and practices

eAppendix 1. Codes for identifying cancer patients based on physician visits and cancer diagnosis and chemotherapy use.

Cancer patients

We identified individuals with cancer based on at least 2 face-to-face outpatient physician visits (evaluation and management codes [E&M] 99201-99215, 99241-99245) with an International Classification of Disease, 9th edition (ICD-9) diagnosis code for cancer (140-209 except 173) or one inpatient admission with a primary ICD-9 diagnosis of cancer.

HCPCS codes for face-to-face office visits:

99201-99215 – office visits or other E&M services
99241-99245 – outpatient visit consultation codes (used through 2010)

We identified patients receiving chemotherapy with a cancer diagnosis code on the claim for one of the cancer sites of interest for which bevacizumab has been approved (colorectum, lung, breast, kidney, brain or ovary).

Chemotherapy use

Any chemotherapy use: J codes: J8999, J7150, J85xx, J86xx, J87xx, J9xxx

Bevacizumab use: J code J9035

International Classification of Disease, 9th Edition codes for cancer sites of interest:

Colon/rectal cancer
153 (colon)
154, 154.0, 154.1, 154.8 (rectum, rectosigmoid junction), excluding 154.2, 154.3 (anal cancer)

Breast cancer
174 (female breast)

Lung cancer
162, 162.2, 162.3, 162.4, 162.5, 162.8, 162.9 (bronchus or lung) excluding 162.0 (trachea)

Renal cell cancer
189, 189.0 (kidney)

Glioblastoma multiforme
191 (brain—note this is nonspecific)

Ovarian cancer
183 (ovary and other uterine adnexa)

We required all patients to have at least 2 outpatient face-to-face office visits at least 30 days apart with an ICD-9 diagnosis of one of the sites of interest.

© 2020 Keating NL et al. *JAMA Network Open.*

eAppendix 2. Assignment of patients to physicians.

1. We first looked at all doctors on the patients' chemotherapy claims for the year, and if they only had one doctor and that doctor had an evaluation and management (E&M) visit with the patient, then they were assigned to the patient. (71% of the patients in 2006)
2. For the patients with more than one doctor on their chemotherapy claims, the doctor with the most E&M visits with the patient was assigned.
3. If there was a tie on E&M visits for the multiple doctors, then we selected the physician based on the position of the specialty in the list below item 6.
4. If the multiple doctors were in the same specialty and were also tied on the number of E&M visits, then we selected the doctor from the chemo claim that was closest to the first date in the year with a cancer diagnosis code. (#2, 3, and 4 account for 25% of the patients in 2006)
5. For the patients with doctors on chemo claims who did not have any E&M visits with the patient, we examined the E&M visits for all doctors of these patients and chose the doctor with the first E&M visit that came on or after the diagnosis date.
6. If there were multiple doctors on the same date, then we selected the physician based on the specialty hierarchy below. (#5 and #6 account for 3% of the patients in 2006)

In case of ties, we used the following hierarchy:

90 medical oncology
83 hematology/oncology
82 hematology
98 gynecologic oncology
16 obstetrics/gynecology
92 radiation oncology
34 urology
04 otolaryngology
11 internal medicine
08 family practice
01 general practice
02 general surgery
33 thoracic surgery

The remaining are quite infrequent, and no clear order… assigned at random)

06 cardiology
29 pulmonary
44 infectious diseases
66 rheumatology
77 vascular surgery
93 emergency medicine

© 2020 Keating NL et al. *JAMA Network Open.*

eFigure. A bipartite network used to construct a unipartite network.

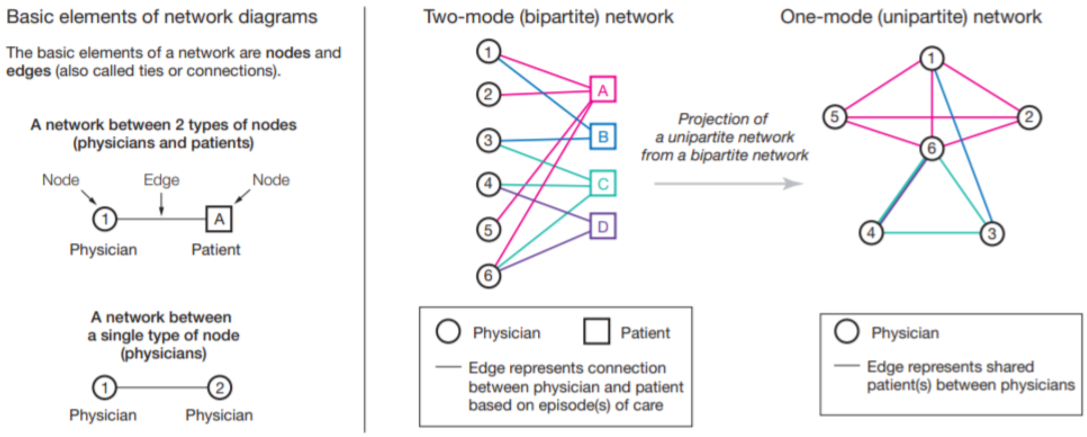

Legend: The figure shows the construction of a projected unipartite network of physicians from a bipartite network of patients and physicians. Doctor-patient relationships are firs identified; the bipartite network is depicted in the center. Next, the unipartite network is derived by identifying the doctors that are connected through shared patients (right). Adapted from Landon et al, JAMA 2012 with permission. Copyright ©2012, American Medical Association.

© 2020 Keating NL et al. *JAMA Network Open.*

eAppendix 3. Assignment of physicians to practices

Each physician was assigned to an oncology practice [identified using tax identification numbers (TINs)] based on the plurality of office visits (evaluation and management codes 99201-99215, 99241-99245) with cancer patients who were receiving chemotherapy. Physicians with equal numbers of patient visits to >1 practice were assigned to the practice with the greatest sum of allowed charges.

© 2020 Keating NL et al. *JAMA Network Open.*
